# Extraction of Metal Ions by Interfacially Active Janus Nanoparticles Supported by Wax Colloidosomes Obtained from Pickering Emulsions

**DOI:** 10.3390/nano12213738

**Published:** 2022-10-25

**Authors:** Oliver Pauli, Andrei Honciuc

**Affiliations:** 1 Institute of Chemistry and Biotechnology, Zurich University of Applied Sciences, Einsiedlerstrasse 31, 8820 Wädenswil, Switzerland; 2 Electroactive Polymers and Plasmochemistry Laboratory, “Petru Poni” Institute of Macromolecular Chemistry, Aleea Gr. Ghica Voda 41A, 700487 Iasi, Romania

**Keywords:** Janus nanoparticles, colloidosomes, metal ion extraction, wastewater treatment, Pickering emulsions

## Abstract

Most common wastewater treatment technologies for ion extraction and recovery rely on pumping wastewater through ion-exchange columns, filled with surface-functionalized polymer microspheres. To avoid the energetically intensive process of pumping large quantities of water through ion-exchange columns, alternative technologies are being developed, such as water-floating membranes containing ligands. In this context, innovative materials could be deployed. Here, we report nanostructured paraffine wax microspheres capable of floating on water, a design based on Pickering emulsion technology, where Janus nanoparticles act both as emulsion stabilizers and as ligand carriers. In the process of emulsification of molten wax in water, followed by cooling, the branched polyethylenimine (bPEI) carrying Janus nanoparticles are trapped at the molten wax/water interface, forming spherical microspheres or colloidosomes decorated with nanoparticles. The paraffine wax colloidosomes stabilized by ligand-carrying Janus nanoparticles are capable of floating on water and show high metal ion extraction capacities towards Cr(VI), Co(II), Ni(II), Cu(II) and Zn(II). In addition, we demonstrate that the ions can be recovered from the colloidosomes and that the colloidosomes can withstand several extraction/recovery cycles with little or no loss in the absorption capacity.

## 1. Introduction

**Scheme 1:**
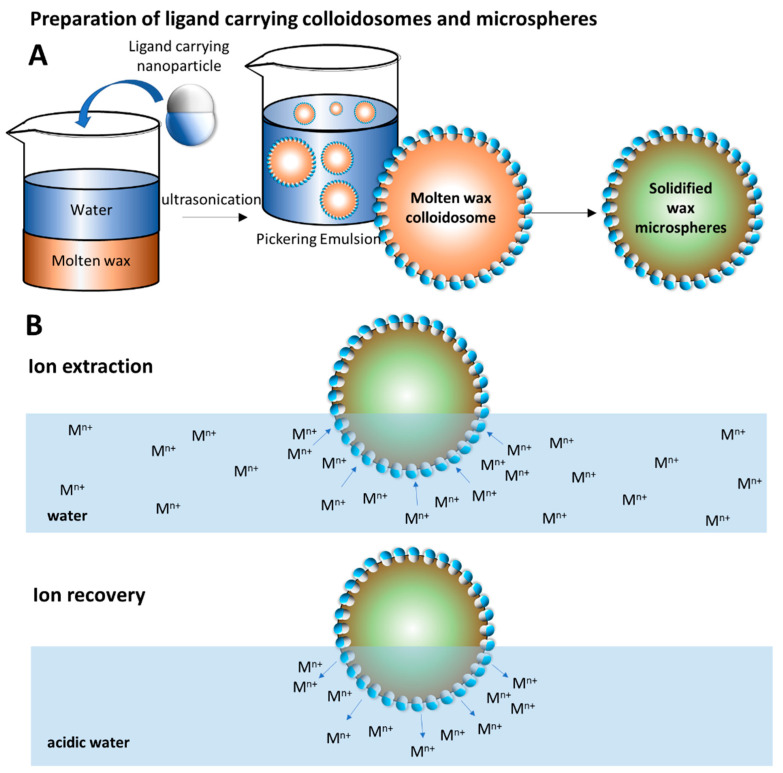
(**A**) Illustration of the methodology proposed for the synthesis of molten wax colloidosomes, and microspheres decorated with ligand-carrying JNPs; (**B**) Principles of the method for employing water-floating ligand-carrying microspheres in ion extraction from wastewater at normal pH and ion recovery in acidic water.

The development of energetically efficient wastewater treatment technologies for the removal of small organic pollutants or heavy metal ions is of paramount importance for maintaining a clean environment and mitigating current water pollution problems. The advantages and drawbacks of various existing technologies for metal ion removal from wastewater, such as chemical precipitation, ion exchange, adsorption, membrane filtration, coagulation and flocculation, flotation, electrochemical treatment, etc., have been extensively reviewed [1]. Finding alternative technologies to the currently existing methods that avoid secondary pollution, generation of solid waste, are energetically efficient and have low operation costs could be achieved by deployment of advanced materials, and this represents a forthcoming challenge for fundamental science and engineering. In this context, for extraction and removal of metal ion pollutants or hydrological mining of noble metals, liquid membranes technologies such as bulk liquid membranes (BLMs), emulsion liquid membranes (ELMs) [2], supported liquid membrane (SLMs) [3] supported ionic liquid membranes (SILM) [4], or polymer inclusion membranes (PIM) [5,6], nanoparticles incorporated into an absorptive film [7], have been proposed as energetically efficient alternatives to classical methods. All these alternative methods involve the interfacial transfer of the metal ions from the water phase into a liquid organic phase or a solid-state polymer containing chelating agents. For example, in ELMs methods, the oil-in-water (o/w) emulsion, with the organic phase containing a chelating agent, is stirred in a reservoir until the ion extraction is completed. For a large interfacial area and efficient extraction, small emulsion droplets are required, however, droplet coalescence and emulsion stability are the limiting factors of the method, which must be resolved [8]. One possible solution to solve the emulsion stability in ELMs is to use the more stable Pickering emulsions, which are emulsions stabilized by nanoparticles or amphiphilic Janus nanoparticles (JNPs) [9,10]. In this case, amphiphilic nanoparticles such as JNPs can play a double role, as emulsion stabilizers in ELMs but also as ligand carriers, whereas ligands can be immobilized on the surface of the nanoparticle by specific surface grafting techniques. In this work we synthesize amphiphilic JNPs, which carry ligands and are also interfacially active, being capable of partitioning at the oil-water interface and are thus able to stabilize Pickering emulsions. JNPs are asymmetric nanoparticles consisting of at least two lobes differing in their chemical composition or surface properties. The contrasting properties between lobes give rise to an intrinsic amphiphilic property resembling molecular surfactants [11,12]. With the help of these ligand-carrying amphiphilic JNPs, we emulsify molten paraffin wax to create o/w emulsions. The proof of concept of this technology is illustrated in the cartoon of Scheme 1. By emulsifying the molten wax in water, colloidosomes are obtained, which have a monolayer of amphiphilic JNPs at their surface. Amphiphilicity is key to the emulsification ability of the JNPs [13]. Colloidosomes can be defined as microcapsules with a shell of nanoparticles, which are obtained by self-assembly of nanoparticles at the interface between two immiscible liquids, most commonly water and oil [14]; these colloidosomes can be employed directly for ion extraction. Although the molten wax colloidosomes cannot preserve their integrity in ion extraction and recovery cycles, upon cooling, the liquid molten paraffin droplets solidify, generating surface nanostructured microspheres by trapping the Janus nanoparticles at the interface. The aim of this work is to show that water-floating wax microspheres decorated with ligand-carrying JNPs, which result from the solidification of wax-in-water Pickering emulsions, can be successfully employed for metal ion extraction, as depicted in Scheme 1. Furthermore, the resulting solid-state colloidosomes/microspheres can be easily regenerated and re-used in many metal ion extraction cycles. The great advantage of using wax colloidosomes over liquid emulsions is that the wax colloidosomes, in absence of stirring, float on the surface of the water which greatly simplifies the collection process, eliminating the need for filtration and further processing steps. The novelty of this work consists in obtaining paraffin microspheres decorated with amphiphilic JNPs that carry ligands and employing them in ion extraction technologies. In addition, their ability to float on water surfaces makes them attractive for the fact that they can be easily collected by water-sweeping barriers and thus minimize the microparticle loss in the water and water pollution with microplastics. This could be a great advantage for the treatment of wastewater, especially in comparison to more energetically intensive techniques based on pumping water through ion exchange columns and can be more practical than the above-mentioned liquid-based floating membrane technologies SLM, ELM and PIM. The collected microspheres from the water surface can be regenerated by the recovery of the absorbed ions in acidic water and can be reutilized in ion extraction, see Scheme 1. To demonstrate this, we use amphiphilic Janus nanoparticles and solid Pickering emulsion for the recovery of Cr(VI), Co(II), Ni(II), Cu(II), Zn(II), which are among the most common heavy metal ions found in wastewater.

## 2. Materials and Methods

### 2.1. Materials

Styrene (St) (>99%), divinylbenzene (DVB) (80%), sodium 4-vinylbenzenesulfonate (NaVBS) (>90%), ammonium persulfate (NH_4_)_2_S_2_O_8_ (APS) (>98%), 2,2′-azobis(2-methylpropionitrile) (AIBN) (>98%), ammonium hydroxide solution (NH_4_·OH) (28%) and basic alumina (Al_2_O_3_) (≥98%), 3-(triethoxysilyl)propionitrile (TESPN) (97%), 3-(triethoxysilyl)propyl-methacrylate (TSPM) (99%), sulfuric acid (H_2_SO_4_) (95.0–98.0%), branched *polyethyleneimine* (bPEI, *M*_n_ ≈ 10,000 by GPC, *M*_w_ ≈ 25,000 by LS) and paraffin wax (mp 53–58 °C) were purchased from Sigma-Aldrich (Buchs, Switzerland). *N,N’*-diisopropylcarbodiimide (DIC) (99%) was purchased from Acros Organics (Basel, Switzerland). St and DVB were passed through basic alumina to remove the stabilizer before usage. AIBN was purified by re-crystallization twice from methanol and stored at −20 °C before usage. Other reagents were used as received. Ultrapure water (UPW; conductivity c = 0.055 µS/cm and resistivity, ρ = 18.2 MΩ cm at 298 K) was obtained from an Arium 611 VF water purification system (Startorius stedim biotech, Aubagne, France), and it was used as the aqueous medium in all experiments.

### 2.2. Synthesis of PS Seed Nanoparticles

The surfactant-free emulsion *co*-polymerization of styrene (St), divinylbenzene (DVB) and sodium vinylbenzenesulfonate (NaVBS) was performed according to a procedure we have previously reported [10,15].

### 2.3. Synthesis and Surface Modification of Janus Nanoparticles

To differentiate between the multiple types of particles and lobe size ratios, the notation JNP-X-Y was adopted, where X is the predominant surface group on the P(3-TSPM) lobe (e.g., CN) and Y is the volume of 3-TSPM/3-TSPCN mixture in mL per 1 g of PS seed NPs.

The synthesis procedure is described in the example of JNP-CN 2 mL. Reagent quantities used for the synthesis of the homologous series (JNP-CN 2 to JNP-CN 4 mL) are given in the Supporting Information (SI).

A suspension of 2 g PS seed NPs in UPW was deoxygenated by bubbling Ar gas under stirring at room temperature (RT). 3 mL 3-TSPM, 1 mL 3-TSPCN and 40 mg AIBN were mixed in a scintillator vial and sonicated for a few seconds to dissolve the AIBN. A total of 11 mL of argonated UPW was added to the mixture, which was then cooled to approx. −40 °C using an ice/acetone bath. The mixture was emulsified by ultrasonication (Branson Sonifier 450, ½ inch processing horn, Branson Ultrasonics Dietzenbach, Dietzenbach, Germany, 2 min at 50% intensity). The monomer-in-water emulsion (which is an o/w emulsion) was added to the dispersion of PS seed NPs using a syringe. The reaction mixture was left to stir at RT for approx. 3 h. 15 droplets of NH_4_OH (30% aq. sol.) were then added to adjust the pH to 9. The polymerization was carried out under an Ar atmosphere while stirring at 70 °C for approx. 12 h. The JNPs were purified by centrifugation/resuspension in ethanol (EtOH) and UPW (three cycles each).

The JNP-CN were then hydrolyzed to JNP-COOH in HCl 2.5 M under reflux for approx. 12 h. The particles were washed by centrifugation/resuspension in UPW until the supernatant had a neutral pH.

### 2.4. Grafting of bPEI to the Janus Nanoparticles

The JNP-bPEI used for the metal ion extraction studies was synthesized using an excess of bPEI and DIC. The quantities of the reagents used for the bPEI loading study are given in the Supplementary Materials.

To a suspension of 2 g JNP-COOH in dry *N,N*-dimethylformamide (DMF), 10 mL DIC was added. The reaction mixture was left to stir under an Ar atmosphere for 15 min. 200 mg bPEI (10% m/m in relation to JNP-COOH) was dissolved in a small volume of DMF and added dropwise. The mixture was left to stir at RT for approx. 12 h. The particles were washed by centrifugation/resuspension, once in a solution of DMF/H_2_O (9:1), twice in EtOH and three times in UPW.

### 2.5. Preparation of Wax Colloidosomes

2500 mg paraffin wax with a melting point of 53–58 °C (C_n_H_2n+2_, n = 24–36 estimated from melting point [16]) was added to a suspension of 250 mg JNP-bPEI in 50 mL UPW. The mixture was heated to 80 °C using a water bath. After the wax was completely molten, the phase-separated mixture was emulsified by sonication (Branson Sonifier 450, ½ inch processing horn, 40 s at 50% intensity). The emulsion was then cooled rapidly using an ice/acetone bath. The solidified wax colloidosomes were filtered off, washed with copious amounts of UPW, and left to dry at RT. The dry colloidosomes were Au-sputtered (Quorum Q150 RS Plus, 20 mA for 30 s), (Quorumtech, Laughton, UK) for characterization by SEM.

### 2.6. Metal Ion Extraction and Recovery

To test the extraction capacity of the synthesized materials, 50 mg JNP-bPEI or 250 mg colloidosomes were suspended in 5 mL of a metal salt solution with *c* = 10 mmol/L at the natural pH of UPW (pH 5.5 ± 0.5). After mechanical shaking at 5000 rpm for 10 min, the samples were left undisturbed for approx. 12 h. The JNP suspensions were centrifuged at 10,000 rpm for 20 min and the supernatant was collected for analysis. The particles were washed through three cycles of centrifugation/resuspension in UPW. The colloidosome samples were filtered (10 µm pore size) and the filtrate was collected for analysis. The colloidosomes were washed with copious amounts of UPW. The supernatant and filtrate obtained from experiments using JNPs and colloidosomes, respectively, were diluted to a metal ion concentration of approx. 50 mg/L and acidified to prevent the formation of metal hydroxides during analysis. Standard solutions for calibration were prepared from the corresponding metal salts in UPW.

The metal ion concentration in the diluted supernatant and filtrate were analyzed using Inductively coupled plasma-optical emission spectrometry (ICP-OES) (ICP-OES 5100, Agilent Technologies, Basel, Switzerland) and compared to the initial concentration. The metal ion extraction capacity *q*_e_ (mg/g) was calculated by:

(1) $$q_{e}=\frac{\left(c_{i}-c_{e}\right)\;V}{m_{P}}$$

where *c*_i_ (mg/L) is the initial concentration, *c*_e_ (mg/L) is the extracted concentration, *V* (L) is the volume of the sample, and *m*_P_ (g) is the dry mass of the sorbent.

The washed JNPs and colloidosomes were then resuspended in 5 mL 0.5% H_2_SO_4_ by means of mechanical shaking at 5000 rpm for 10 min. To aid redispersion, the JNP samples were also sonicated for 10 min. The samples were then left undisturbed for approx. 12 h. The particles were separated from the aqueous medium as described previously. Supernatant and filtrate were analyzed using ICP-OES, and the metal ion recovery capacity *q*_r_ (mg/g) was calculated by:

(2) $$q_{r}=\frac{c_{r}\;V}{m_{P}}$$

where *c*_r_ (mg/L) is the concentration of metal ions recovered from the particles, *V* (L) is the volume of the sample, and *m*_P_ (mg) is the dry mass of the particles.

The JNPs and colloidosomes were washed as described previously, and the entire procedure was repeated at least three times. All ICP-OES measurements were performed in triplicate.

### 2.7. Statistical Analysis of the Data

To have a proper understanding of the reproducibility of the extraction and recovery of the ions by both the JNPs and wax microspheres, we have repeated all ICP-OES measurements at least three times. For each extraction step, we have repeated the procedure three times; in this work, the average value of the obtained extraction capacity *q*_e_ is given in the graphics together with the standard deviation obtained for each measurement. The same statistical treatment was done for measurements of the ion recovery capacity *q*_r_ of the materials tested, i.e., JNPs and microspheres. The total error of the measurement *σ*_total_ was considered to be the total standard deviation from two sources, namely the error in the ICP-OES measurements *σ*_measurement_ and that of extraction or recovery experiments *σ*_experiment_:

$$\sigma_{\mathrm{total}}=\sqrt{{\left(\sigma_{\mathrm{measurement}}\right)}^{2}+{\left(\sigma_{\mathrm{experiment}}\right)}^{2}}$$

To determine if the means of the two groups are significantly different, a standard independent (two-sample) *t*-test was used.

## 3. Results and Discussions

### 3.1. Synthesis and Functionalization of Janus Nanoparticles (JNPs)

**Scheme 2:**
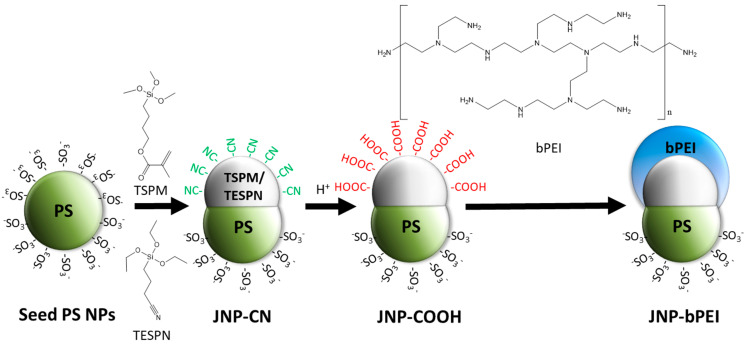
Reaction scheme depicting the synthesis of seed PS JNPs, synthesis of JNP-CN and their conversion into JNP-COOH and JNP-bPEI.

Polystyrene seed PS seed-NPs with an average diameter of 305 ± 3 nm were synthesized using surfactant-free emulsion polymerization, according to Scheme 2. Onto these PS seed NPs, a second lobe was grown through a seeded emulsion co-polymerization of 3-(trimethoxysilyl)propyl-methacrylate (TSPM) and 3-cyanopropyltriethoxysilane (TESPN) monomers followed by phase separation, see Scheme 2, resulting in Janus nanoparticles bearing -CN groups on the second lobe (JNPs-CN) according to previously reported methods published by our group [10,15]. The size of the second lobe relative to the PS lobe could be adjusted by varying the volume of TSPM and TESPN monomers in relation to a reference weight of 1 g of PS NPs. Thus, a homologous series of JNPs-CN was created by using 2 mL, 3 mL and 4 mL of monomers per 1 g of PS NPs. The nitrile groups on the surface of the second Janus lobe were hydrolyzed under reflux in hydrochloric acid, resulting in Janus nanoparticles bearing carboxylic acid surface functional groups (JNPs-COOH), as shown in Scheme 2. The conversion was confirmed through IR spectrometry (Figure S1) and the pH-dependent measurement of the surface zeta potential in Figure S2 and the comparative values of the homologous series of JNPs in Table S1. Through SEM imaging it was confirmed that the appearance of the particles was not altered by the acid treatment (Figure S3), a testament to the good chemical stability of polymeric JNPs.

### 3.2. Selective Grafting of bPEI on One Janus Lobe

*N,N’*-Diisopropylcarbodiimide (DIC), a coupling agent commonly employed in peptide chemistry, was used to selectively graft branched polyethylenimine (bPEI, *M*_n_ ≈ 10,000 by GPC) on the second Janus lobe bearing carboxyl functional groups JNPs-COOH, according to Scheme 2. This was the only reaction where the main solvent was not water. The success of the reaction was confirmed through IR spectrometry (Figure S1) and the pH-dependent measurement of the surface zeta potential (Figure S4). The appearance of the JNPs under the SEM remained unchanged (Figure S5) and a corona of bPEI on the TSPM/TESPN Janus lobe is too thin to be visible.

### 3.3. Interfacial Activity of JNP-bPEI Homologous Series

The interfacial activity of JNP-bPEI at the heptane/water interface was determined using the pendant drop method (OCA 25, DataPhysics). A droplet of an aqueous suspension of JNPs was formed in n-heptane and the curvature of the droplet fitted to the Young–Laplace equation. The interfacial tension (IFT) of a pure heptane/water interface at 25 °C is γ = 50.71 mN·m^−1^ [17]. In the presence of the JNPs-bPEI, the IFT decreases vs. time due to interfacial adsorption. In Figure S6 it is shown that the interfacial activity, as judged by the lowest values of the IFT reached in the plateau, is significantly larger at lower pH values, which can be explained by the protonation of the bPEI grafted on the second Janus lobe. With the protonation of the bPEI, the surface of the second Janus lobe becomes more polar and produces a good amphiphilic contrast to the less polar PS lobe.

**Figure 1:**
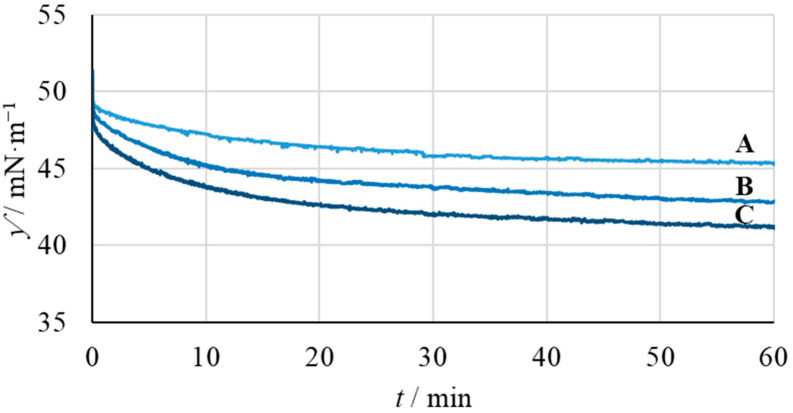
The IFT evolution vs. time of the heptane/water interface in the presence of the Janus nanoparticles (50 mg/mL in the aqueous phase) of the homologous series (A) JNP-bPEI 2 mL, (B) JNP-bPEI 3 mL, (C) JNP-bPEI 4 mL.

Furthermore, we investigated the influence of the Janus lobe ratio on the interfacial activity of the JNP-bPEI. The influence of the lobe size ratio on the interfacial activity of the particles is demonstrated in Figure 1. The IFT of all suspensions starts out ±1 mN·m^−1^ of the pristine heptane/water IFT value and then slowly declines as the particles reach the interface. This also indicates the absence of fast-acting molecular surfactants. It is apparent that increasing the size of the hydrophilic bPEI-modified P(3-TSPM) lobe increases the interfacial activity of the particles (compare curves A, B and C in Figure 1). A similar observation has been reported for similar JNPs by Wu et al. [10,18] and was attributed to the hydrophilic-lyophilic balance of the JNPs, which is also called the Janus balance [19].

Furthermore, the IFT is greatly affected by JNP-bPEI concentration, with a minimum value of ≈ 33 mN/m for a concentration of 300 mg/mL, see Figure S7.

### 3.4. Preparation of Wax Colloidosomes

**Figure 2:**
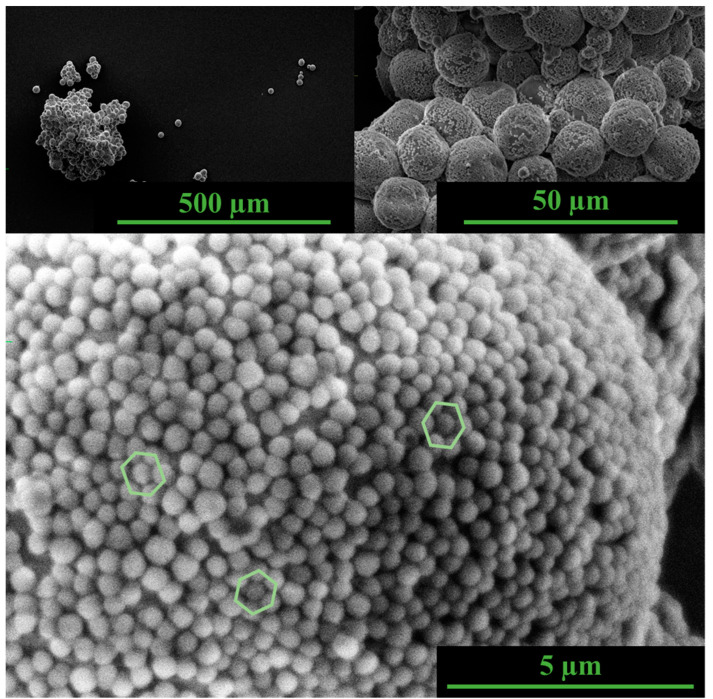
Wax colloidosomes obtained with JNP-bPEI 4 mL.

Colloidosomes consisting of paraffin wax (melting temperature, T_m_ = 53–57 °C) and 10% m/m bPEI-modified JNPs were prepared by emulsifying molten wax in an aqueous suspension of JNPs through sonication, followed by rapid cooling. The washed colloidosomes were characterized using SEM (Figure 2). The diameters of the colloidosomes obtained by using the homologous series of JNPs (JNP-bPEI 2 mL to JNP-bPEI 4 mL) were not significantly different (Figure S8). On average, the diameter of the colloidosomes is *d* = 15.8 ± 0.3 µm. Figure S9 also shows the qualitative difference in the packing densities of the JNP-bPEI 4 mL vs. JNP-bPEI 2 mL on the surface of the wax colloidosome.

### 3.5. Extraction of Metal Ions by JNPs

**Figure 3:**
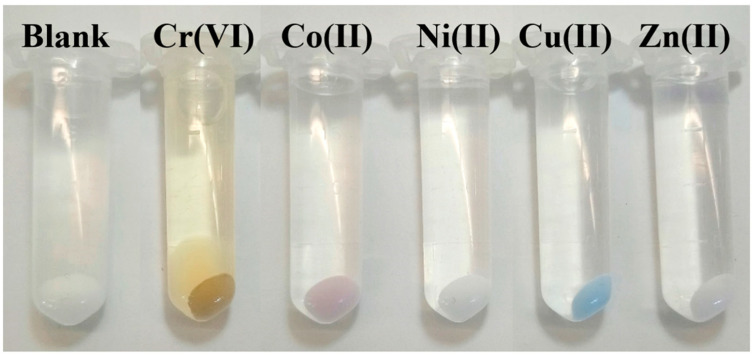
Photograph of the JNP-bPEI 2 mL after absorption of Cr (VI), Co(II), Ni(II), Cu(II), Zn(II) and centrifugation.

JNP-bPEI 2 mL was employed in the extraction of metal ions: Cr (VI), Co(II), Ni(II), Cu(II) and Zn(II). The procedure consisted of adding 10 mg/mL JNP-bPEI to a metal ion solution with a concentration of 10 μmol/mL, the conditions were kept constant throughout all ion extractions as described in the experimental section. The photograph in Figure 3 shows the color of JNP-bPEI 2 mL, after extraction/absorption of Cr (VI), Co(II), Ni(II), Cu(II) and Zn(II). Furthermore, to quantitatively determine the absorption capacity for each metal ion, i.e., metal ion load, the concentrations of metal ions in the supernatant were analyzed using Inductively Coupled Plasma-Optical Emission Spectrometry (ICP-OES) and compared to the initial concentration. The metal ion extraction capacity *q*_e_ (mg/g) was calculated with the formula given by the Equation (1) [20].

**Figure 4:**
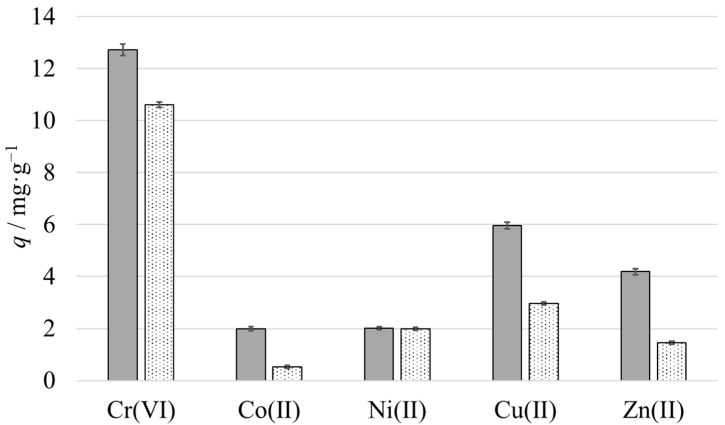
Graph showing the metal ion extraction capacity *q*_e_ (solid bars) and the metal ion recovery capacity *q*_r_ (dotted bars). Error bars represent ± 1 σ.

Figure 3 shows the color change of the particles occurring upon the absorption of certain metal ions, and Figure 4 shows the measured metal ion extraction capacity *q*_e_ of the JNP-bPEI 2 mL. Furthermore, we have determined the number of metal ions that can be recovered, extracted from the JNP-bPEI 2 mL. The recovery was performed in acidic conditions according to the procedures described in the experimental section. The supernatant was analyzed using ICP-OES, and the metal ion recovery capacity *q*_r_ (mg/g) was calculated with formula given by the Equation (2).

To test the metal ion absorption capacity and recovery by JNPs with multiple duty cycles, the JNPs were washed as described previously, and the entire procedure was repeated up two or five times for Cu(II). After an initial decrease of roughly 25% after the first cycle, the extraction/recovery capacity of JNP-bPEI 2 mL remains unchanged for at least four subsequent cycles, Figure S10.

### 3.6. Metal Ion Extraction Performance in the Homologous Series of JNPs

The metal ion extraction and recovery performance in the homologous series of JNPs, with the varying Janus lobe size was investigated for Cr(IV) and Cu(II). One way to indirectly prove the presence of bPEI on the P(3-TSPM) lobe is by increasing its surface area and observing the effect on the metal ion extraction capacity. In the homologous series of JNP-bPEI 2 mL, 3 mL, 4 mL, by increasing the TSPM/TESPN lobe size while the PS lobe remains unchanged, the surface area of the bPEI covered TSPM/TESPN lobe also increases, thus the extraction capacity of the JNPs should also increase. To quantitatively assess this, due to the fact that in the extraction/recovery experiments we have used the same mass of particles, see Table S2, we must be careful to consider the mass of a single particle also increases with an increase in the TSPM/TESPN lobe, which means that fewer nanoparticles will be present in a given mass of JNPs used for extraction. A normalization procedure was devised to account for this effect. Since the average volumes of the individual lobes *V*_PS_, *V*_TSPM_ and their average densities *ρ*_PS_ = 1.03 g/mL, *ρ*_TSPM/TESPN_ = 1.07 g/mL are known, the average mass of a single particle can be calculated according to the following equation:

(3) $$m_{\mathrm{JNP}}=V_{\mathrm{PS}}\times \rho_{\mathrm{PS}}+V_{\mathrm{TSPM}/\mathrm{TESPN}}\times \rho_{\mathrm{TSPM}/\mathrm{TESPN}}$$

From this, the approximate number of JNPs in a given dry mass of particles *m*_w_ can be calculated using the following equation:

(4) $$N_{\mathrm{JNP}}=\frac{m_{w}}{m_{\mathrm{JNP}}}$$

To make the trends of *q*_e_ and *q*_r_ of the homologous series visible, the *m*_w_ values were normalized for an arbitrary particle number of 10^12^ and by plugging in the obtained values in Equations (1) and (2) the normalized metal ion extraction *q*_Ne_ and recovery *q*_Nr_ efficiencies are obtained. The un-normalized and normalized values with Cr(VI) and Cu(II) are depicted in Figure S11 and Figure S12. Normalized values indicate a significant increase in the extraction and recovery capacities with increasing TSPM/TESPN lobe size in the homologous series JNP-bPEI 2 mL, 3 mL and 4 mL. The extraction and recovery capacities of the tested homologous series are listed in Table S2. In comparison, Tan et al.^16^ reported *q*_e_ ≈ 32 mg/g for both Cu(II) and Cr(VI) for their PEI-PS nanoparticles. This is a factor of 5.3 and 2.1 higher than the values found during this study for Cu(II) and Cr(VI), respectively. One possible explanation could be the smaller size of PEI-PS as compared to JNP-bPEI, which results in a higher surface area. Furthermore, PEI-PS is completely covered in bPEI, while only half of JNP-bPEI is covered.

It is instructive to compare the maximum ion uptake obtained for the JNP-bPEI (where the bPEI is a multidentate ligand brush or corona), with the maximum ion load of other micro- and nanoparticles carrying other types of ligands, immobilized in different ways, capable of forming chemical or physical bonds with the metal ion. Among these, most noteworthy are ion imprinted polymer (IIP) micro- and nanoparticles (with ligands immobilized in the bulk of the polymer, capable of chemical bonding with the metal ion), core-shell micro- and nanoparticles whereas the shell is constituted of IIPs, micro- and nanoparticles carrying surface ligands, micro- and nanoparticles carrying anionic functional groups such as ion-exchangers, or agro-based biomasses (capable of interacting with metal ions via physical bonds), etc. Judging purely by the number of ligands a particle can carry, the metal ion uptake capacity for each technology is also expected to decrease with the decrease in ligand carrying capacity of the particulate material, in the following order: IIPs > core-shell IIPs surface immobilized ligands > surface immobilized ligands and ligand brushes > ion-exchangers and biomasses.

For example, the reported ion uptake capacity by JNPs for Zn(II), 4 mg/g, see Figure 4, is higher than the non-chelating physical adsorbing biomasses, whereas the reported maximum ion uptake capacity of biomasses by biosorption of Zn(II) was found to be around 1.688 mg/g for eucalyptus bark, 1.028 mg/g for mango bark and 0.45 mg/g for pineapple fruit peel at a particle size of 0.5 mm [21]. On the other hand, it is expected that the ion-imprinted polymer nanoparticles (IIPs-NPs) offer the best ion uptake capacity, whereas, for the Co(II) for example, the maximum loading capacities reported ranged between 78.3 to 96.6 mg/g by core-shell nanoparticles obtained by constructing an IIP shell of Co-polyacrylamide onto SiO_2_ nanoparticles [22] and 74 mg/g by an IIP shell of Co-dithizone/poly(methacrylic)acid [23] onto magnetic Fe_3_O_4_ core nanoparticles. On the other hand composite chelating IIPs-NPs constituted by a shell of polyamidoxime and a silica core PAO/SiO_2_, showed a maximum Cu(II) ion uptake of 10 mg/g [24], which is comparable to the Cu(II) ion uptake by JNP-bPEI of 6 mg/g, see Figure 4. Even so, the Ni(II) ion absorption capacity of an ion-imprinted polymer (IIP) obtained by copolymerization of a Ni-dithizone complex with 4-vinylpyridine and ethyleneglycoldimethacrylate (EGDMA) is 1.3 mg/g [25] The value found for Ni(II) for JNP-bPEI in this study is higher by a factor 1.5, see Figure 4. Tan et al. [20] have reported ion extraction capacities between 20 and 40 mg/g for Cu(II), Co(II), Ni(II) and Cr(VI) for polystyrene nanoparticles modified as in the current case with a bPEI ligand brush or corona Thus, it can be concluded that the JNP-bPEI exhibit a good ion extraction or ion-uptake capacity, comparable to the extraction capacity of other ligand carrying micro- and nanoparticles obtained with other technologies, in the expected performance range.

### 3.7. Metal Ion Extraction by JNPs Supported by Wax Colloidosomes

**Figure 5:**
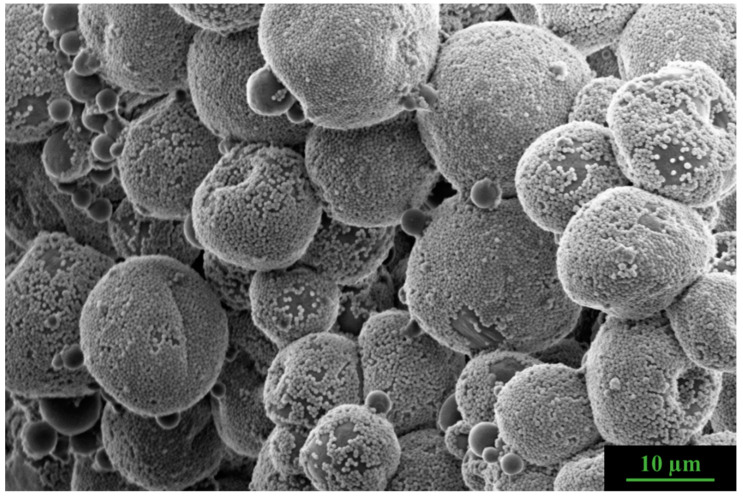
SEM image of the wax colloidosomes decorated with JNP-bPEI 3 mL, employed in interfacial extraction of metal ions.

The wax colloidosomes obtained by stabilizing the molten wax with JNP-bPEI were also employed in the metal ion extraction and recovery cycles, see Figure 5.

**Figure 6:**
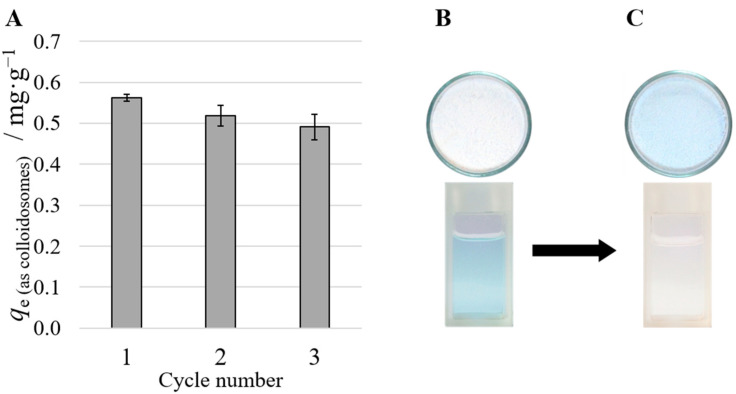
(**A**) Extraction performance of Cu(II) by wax colloidosomes stabilized with JNP-bPEI 3 mL after 3 consecutive extraction/recovery cycles. (B,C) Qualitative proof of the interfacial absorption/extraction of Cu(II) ions from water by floating JNP-bPEI decorated wax colloidosomes. To accurately represent the colors of the solution and the colloidosomes, they were photographed separately. (**B**) A layer of white, pristine (unwetted) colloidosomes with a mass of 6 g is floating on the surface of 10 mL of a blue 10 mM Cu(II) sulfate solution. (**C**) The sample was mechanically shaken and then left to separate overnight. After extraction of the solution with an appropriate amount of colloidosomes, the former becomes colorless while the latter appears bright blue.

As already mentioned, the great advantage of these materials, JNPs and JNP decorated colloidosomes, is the ability to employ them in interfacial extraction technologies, with the main benefit of ease of deployment, collection and recovery from the water’s surface and re-deployment on the surface of the water for the subsequent metal ion extraction/recovery cycles. To test the versatility of these JNPs-decorated wax colloidosomes in such technologies, we have deployed them for Cu(II) extraction. The graph in Figure 6A shows the extraction efficiencies *q*_e_ for three consecutive cycles from fresh 10 mM Cu(II) solutions, as described in the experimental section. In a different experiment, two portions of 6 g of colloidosomes were used to extract Cu(II) from the same 10 mL solution with an initial concentration of 10 mM. After the first extraction cycle, the concentration of copper in the solution was reduced to 4 mM and after the second extraction cycle, the concentration of Cu(II) decreased further to 0.3 mM, see Figure 6B,C. The numbers are in good agreement with the extraction capacities *q*_e_ calculated by the “differential concentration” method: (*q*_e_ ≈ 0.55 mg/g Colloidosome ≈ 8.7 µmol/g ≈ 52 µmol Cu(II)/6 g Colloidosome; 10 mL soln. with c = 10 mM → 100 µmol Cu(II)). The photographs in Figure 6B,C clearly demonstrate that a floating layer of 6 g of wax colloidosomes decorated with JNP-bPEI 3 mL, placed above a water solution of Cu(II) ions, and after brief shaking to ensure the wetting of the nanoparticles, is capable of almost complete interfacial extraction of the ions overnight. Next, the metal-loaded colloidosomes can be easily collected from the water’s surface and dried; see the color change from white to blue of colloidosomes, before and after the Cu(II) metal ion extraction in the photographs of Figure 6B,C. The experiments were limited only to extraction, to demonstrate the working principle and versatility of wax colloidosomes and a recovery cycle for Cu(II) was not made.

It is important to note that the amphiphilicity and the interfacial activity of JNP-bPEI are important for both emulsification and to ensure the proper orientation of the Janus lobes at the surface of wax colloidosomes. In this case, good amphiphilic behavior ensures that the hydrophobic PS Janus lobe is oriented toward the wax surface, while the hydrophilic lobe covered with bPEI provides a large surface area for capturing metal ions. Therefore, this aspect is crucial in designing water-floating colloidosomes and employing them in the interfacial extraction of metal ions.

**Figure 7:**
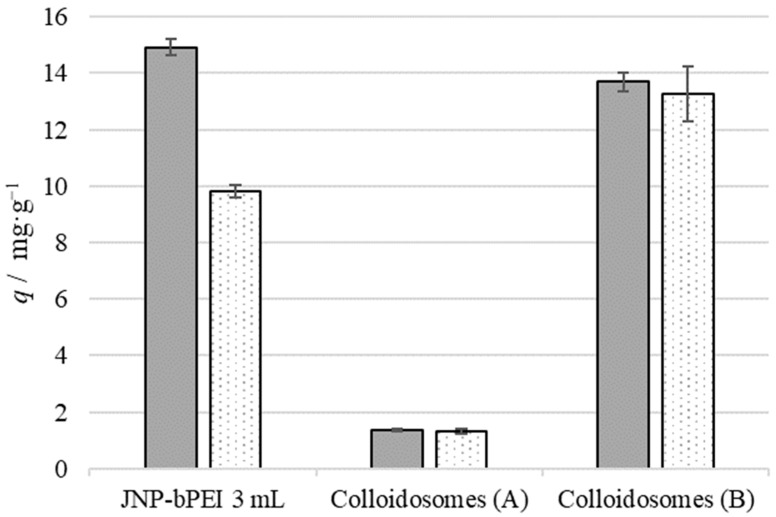
Comparison of *q*_e_ (filled bars) and *q*_r_ (dotted bars) between JNP-bPEI 3 mL and the colloidosomes that were prepared with it. *q* of Colloidosomes (**A**) was calculated using the total mass of the colloidosomes. *q* of Colloidosomes (**B**) was calculated using the approx. mass of JNP-bPEI in the colloidosomes.

Furthermore, for a quantitative interpretation of the absorption capacity, both JNP-bPEI and JNP-bPEI-covered wax colloidosomes were employed in the extraction and recovery of the Cr(VI) comparative study. Thus, for the preparation of wax colloidosomes, 50 mg JNP-bPEI and 500 mg wax were used. Since not all colloidosome surfaces are fully covered it is reasonable to assume that the entire quantity of JNPs is incorporated into the surface of the wax, which for colloidosomes with an average radius of 15 μm, results in a mass fraction of *m*_JNP_/*m*_Colloidosome_ = 10%. Calculating *q*_e_ and *q*_r_ based on that assumption, it becomes apparent that incorporation of the JNP-bPEI into wax does not hinder their ability to interact with the ions, see Figure 7. It is important to note that the colloidosome samples were left on a mechanical shaker at 150 rpm overnight to ensure the complete wetting of the surfaces. The low density and slight hydrophobicity of the colloidosomes would otherwise prevent the wetting of the entire material and falsify the results. For demonstrative purposes, Figure S13 shows a sequence of images taken at 10 min intervals from a sample of 200 mg colloidosomes shaken in 5 mL aqueous solution containing Cr(VI) with a concentration of 10 mmol/L, and left to sit undisturbed. After 30 min, the wax colloidosomes can be found floating on the solution surface with complete separation from the liquid phase.

## 4. Conclusions

It was shown that JNP-bPEI exhibits both of the desired characteristics: amphiphilicity and capability to absorb metal ions. We have demonstrated that the amphiphilicity of JNPs is key to enabling new ion extraction and recovery technologies. For example, we have demonstrated that amphiphilic JNP-bPEI can operate as (a) standalone ion-extraction agents, capable of absorbing metal ions from water and then transporting these to the surface where they could be collected and (b) as emulsifiers of molten wax to generate a new type of carriers capable of floating on the surface of water but also of metal ion extraction. There are, however, still multiple aspects left to explore. The kinetics of the extraction and recovery processes could be assessed by analyzing the metal content of the solutions as a function of time. With the optimized extraction times, the recyclability experiments using Cr(VI) could be repeated to see if the capacity still degrades. Different metal ions such as Ni(II) or Co(II) could be used to gather more data concerning the extraction and recovery capacities. The selectivity towards certain ions could also be determined—an important parameter when it comes to real-world applications. In future works, the long-term stability of JNP-bPEI-covered wax colloidosomes should be tested. The colloidosomes are only viable carriers if the nanoparticles stay attached to their surfaces even after prolonged agitation. A scale-up of the preparation procedure should also be attempted, with regard to manufacturing at an industrial scale.

## Data Availability

All data is available upon request from the corresponding author.

## Supplementary Materials

The following supporting information can be downloaded at: https://www.mdpi.com/article/10.3390/nano12213738/s1. Figure S1: FTIR spectra of polystyrene seed nanoparticles, JNP-CN, JNP-COOH and JNP-bPEI, Figure S2: Zeta potential with pH for JNP-CN and JNP-COOH, Figure S3: SEM images of JNP-CN and JNP-COOH, Figure S4: Zeta potential with pH for JNP-bPEI, Figure S5: SEM images of the homologous series of JNPs funcionalized with bPEI, Table S1: Zeta potential of the homologous series of JNP-CN, JNP-COOH and JNP-bPEI, Figure S6: Evolution of the interfactial tension of heptane/water in time in the presence of JNP-bPEI with pH, Figure S7: Interfacial tension of heptane/water in time as a function of JNP-bPEI concentration in water, Figure S8: SEM images of wax colloidosomes prepared with JNP-bPEI 2–4 mL, Figure S9: SEM images of colloidosomes, Figure S10: Performance of JNP-bPEI 2 mL after five consecutive extraction and recovery cycles for Cu(II), Figure S11: Regular and number-normalized Cr(VI) extraction and recovery values for JNP-bPEI 2–4 mL, Figure S12: Regular and number-normalized Cu(II) extraction and recovery values for JNP-bPEI 2–4 mL, Table S2: Metal ion extraction and recovery capacities of the homologous series of JNP-bPEI, Figure S13: Image sequence of colloidosome flotation and images of filtered colloidosomes.
