# Supplementary material for: Extraction of Metal Ions by Interfacially Active Janus Nanoparticles Supported by Wax Colloidosomes Obtained from Pickering Emulsions
Source: Nanomaterials (Basel). 2022 Oct 25;12(21):3738. doi: 10.3390/nano12213738 (PMC9654599; doi:10.3390/nano12213738)
Supplement: Supplementary file 1 [file nanomaterials-12-03738-s001.zip › nanomaterials-1929366-supplementary.pdf]

nanomaterials

Article

Extraction of Metal Ions by Interfacially Active Janus Nanoparticles Supported by Wax Colloidosomes Obtained from Pickering Emulsions

Oliver Pauli [1] and Andrei Honciuc [2,*]

1 Institute of Chemistry and Biotechnology, Zurich University of Applied Sciences, Einsiedlerstrasse 31, 8820 Wädenswil, Switzerland
2 Electroactive Polymers and Plasmochemistry Laboratory, "Petru Poni" Institute of Macromolecular Chemistry, Aleea Gr. Ghica Voda 41A, 700487 Iasi, Romania
* Correspondence: honciuc.andrei@icmpp.ro

Reagent quantities for the Synthesis of JNP-CN

For JNP-CN 2 mL, 2 g PS Seed, 3 mL 3-TSPM, 1 mL 3-TESPN and 40 mg AIBN were used. The quantities of AIBN, 3-TSPM and 3-TESPN were scaled by a factor 1.5 and 2 respectively to synthesize JNP-CN 3 mL and 4 mL.

Reagent quantities for the Synthesis of JNP-bPEI

For JNP-bPEI 2 mL, 2 g JNP-COOH, 3 mL DIC and 50 mg bPEI in a total of 45 mL DMF were used. The quantities of DIC and bPEI were scaled by a factor 1.5 and 2 respectively to synthesize JNP-bPEI 3 mL and 4 mL.

Additional Figures and Tables mentioned in the Publication

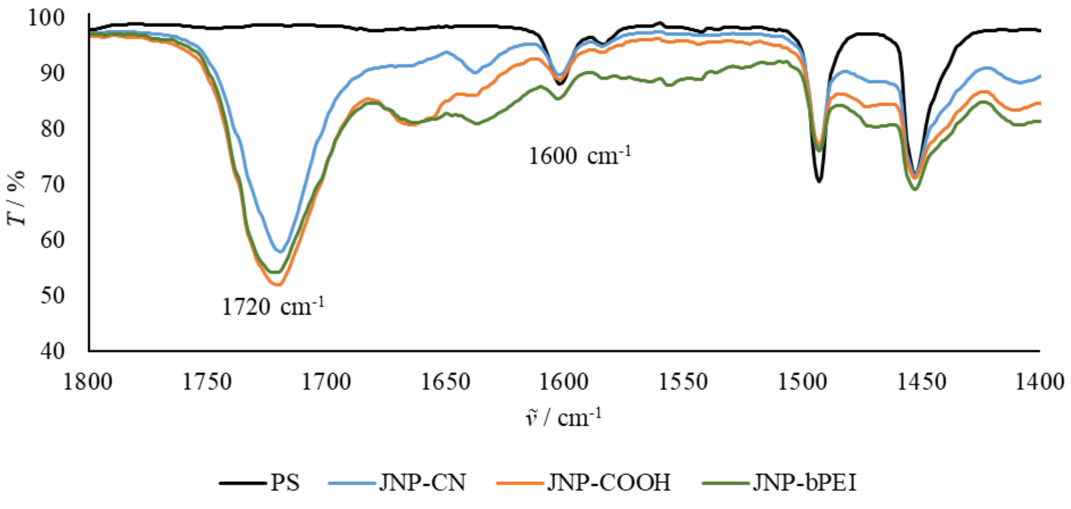

Figure S1. FTIR spectra of polystyrene seed nanoparticles (PS), JNP-CN, JNP-COOH and JNP-bPEI, normalized to the aromatic C-H out of plane deformation vibration of polystyrene at 698 cm^{-1}. The growth of a second lobe containing 3-TSPM on the seed nanoparticle causes the strong carbonyl stretching (C=O st) vibration at 1720 cm^{-1}. Subsequent hydrolysis of the nitrile groups to carboxylic acids leads to a broadening of the C=O st vibration band. Finally, the addition of branched PEI (itself a polymer) leads to stronger absorbance in the N-H bending vibration band around 1600 cm^{-1}.

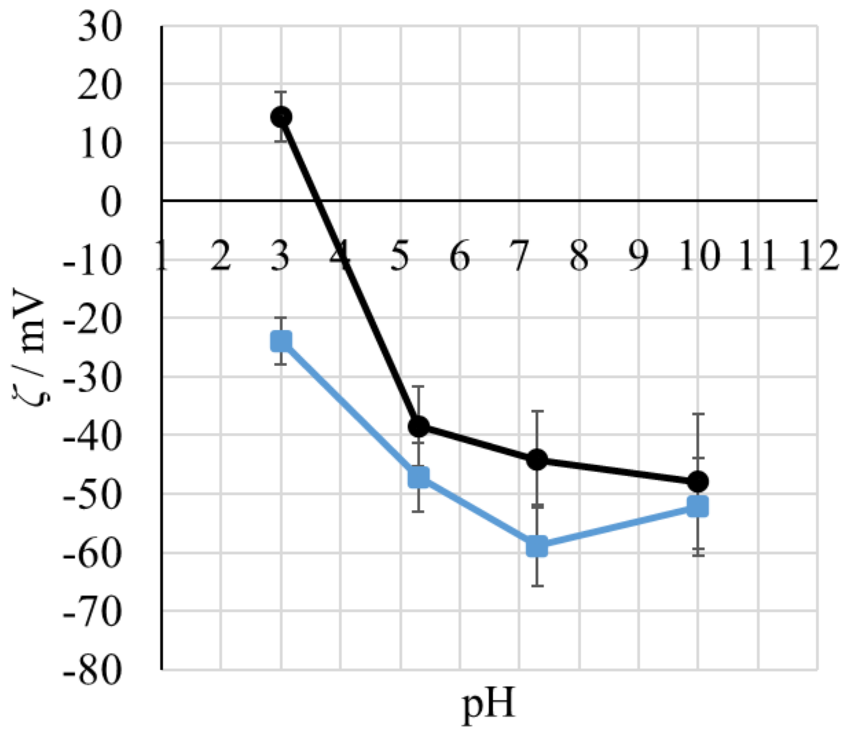

Figure S2. Zeta potential with pH for the TESPN with CN surface functional groups (black Line) and with COOH surface functional groups (blue Line).

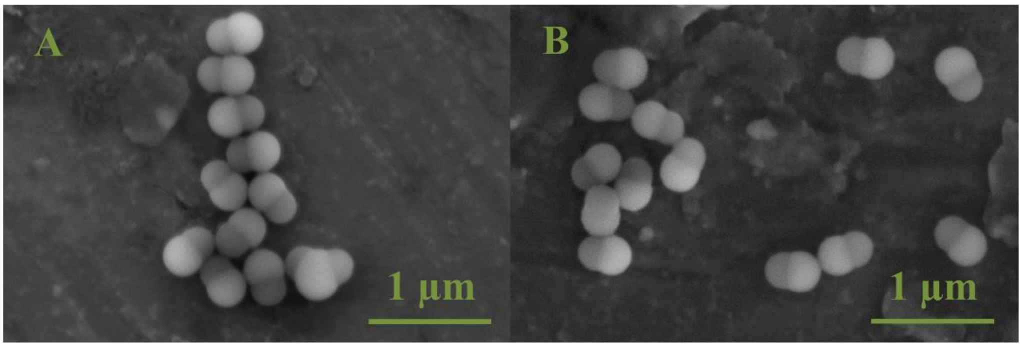

Figure S3. SEM images of (A) JNPs-CN and (B) JNPs-COOH.

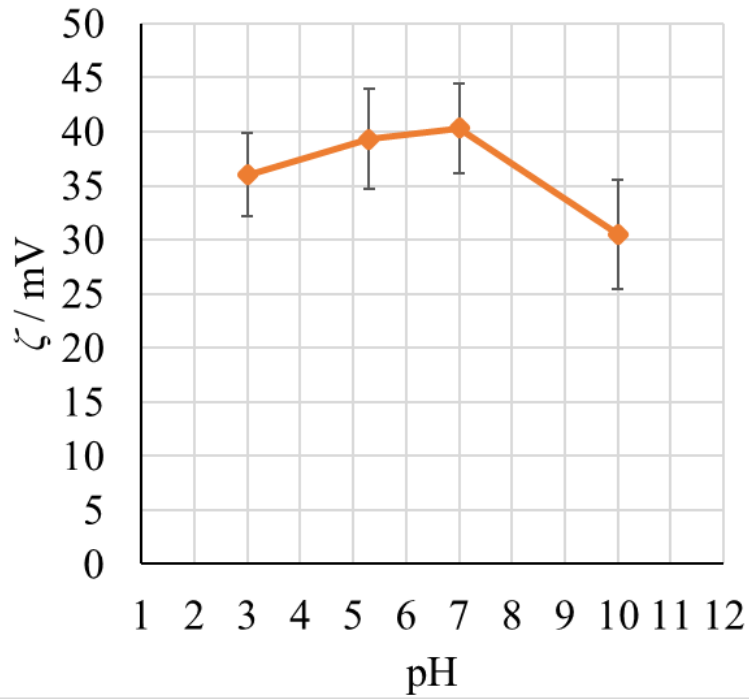

Figure S4. Zeta potential of JNP-bPEI with pH.

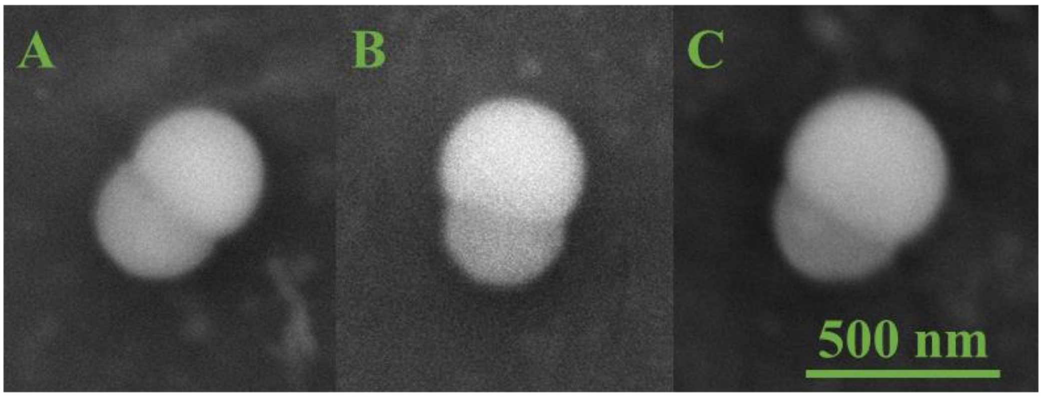

Figure S5. SEM images of the homologous series of JNPs functionalized with bPEI: (A) JNP-bPEI 2 mL, (B) JNP-bPEI 3 mL, (C) JNP-bPEI 4 mL.

Table S1. Zeta potential of the homologous series of JNP-CN, JNP-COOH and JNP-bPEI at pH=5.5 and mass concentration of 0.01 mg/mL.

Type	ζ / mV
JNP-CN 2 mL	-50.5 ± 7.0
JNP-CN 3 mL	-41.9 ± 7.4
JNP-CN 4 mL	-44.5 ± 6.1
JNP-COOH 2 mL	-54.7 ± 5.4
JNP-COOH 3 mL	-54.9 ± 6.1
JNP-COOH 4 mL	-47.5 ± 6.1
JNP-bPEI 2 mL	50.8 ± 6.1
JNP-bPEI 3 mL	51.8 ± 6.1
JNP-bPEI 4 mL	50.4 ± 5.6

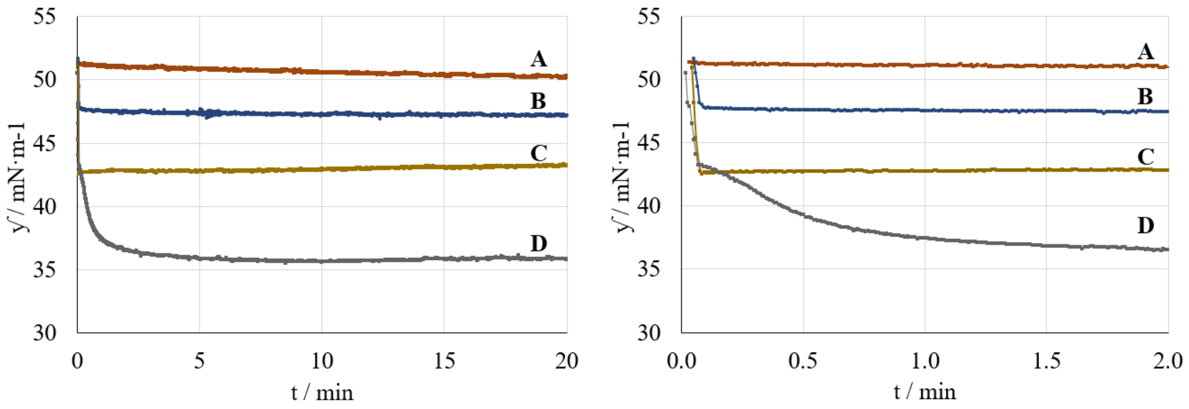

Figure S6. Evolution of the interfacial tension of heptane/water in time in the presence of JNP-bPEI 2 mL at 10 mg/mL in the water phase, demonstrating their interfacial activity: (A) in UPW, pH 6; (B) pH 5; (C) pH 2.5; (D) in HCl 0.5%, pH 1.5. The graph on the right shows a zoom of the first two minutes.

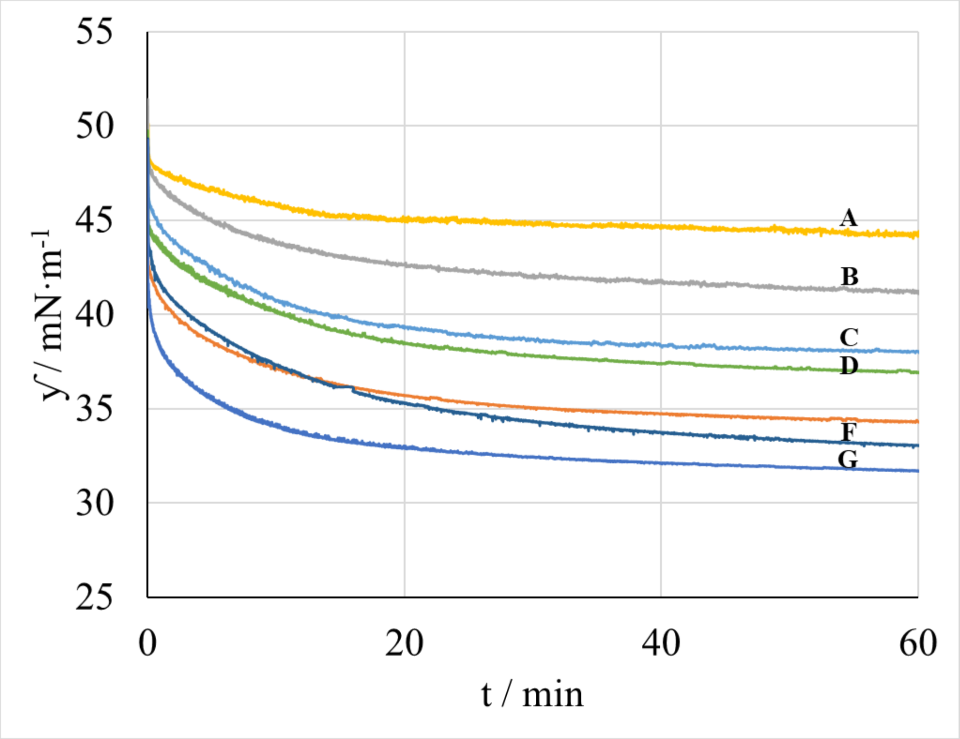

Figure S7. IFT of the heptane/water interfaces vs. time, function of JNP-bPEI concentration in water: (A) 25 mg/mL (B) 50 mg/mL, (C) 100 mg/mL (D) 150 mg/mL (E) 200 mg/mL (F) 250 mg/mL (G) 300 mg/mL.

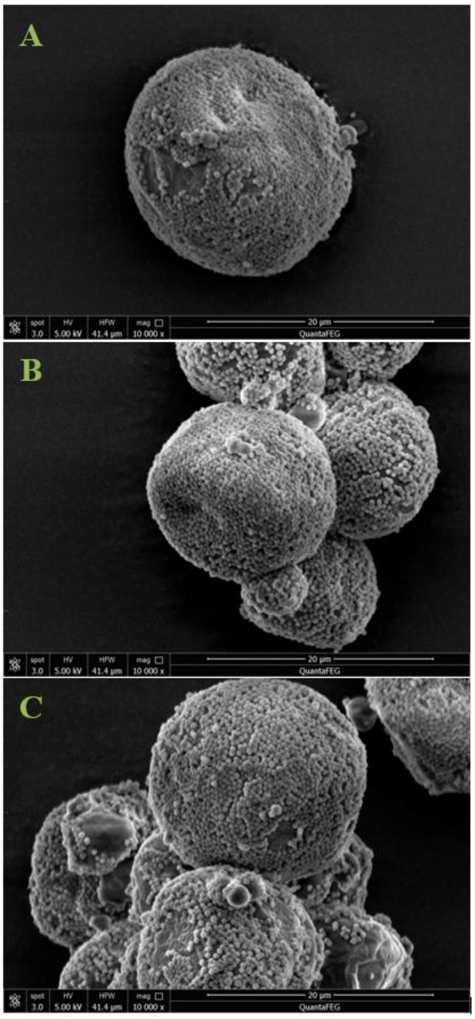

Figure S8. SEM images of wax colloidosomes prepared with (A) JNP-bPEI 2 mL, (B) JNP-bPEI 3 mL, (C) JNP-bPEI 4 mL. The wax colloidosomes were sputtered with 4 nm Au.

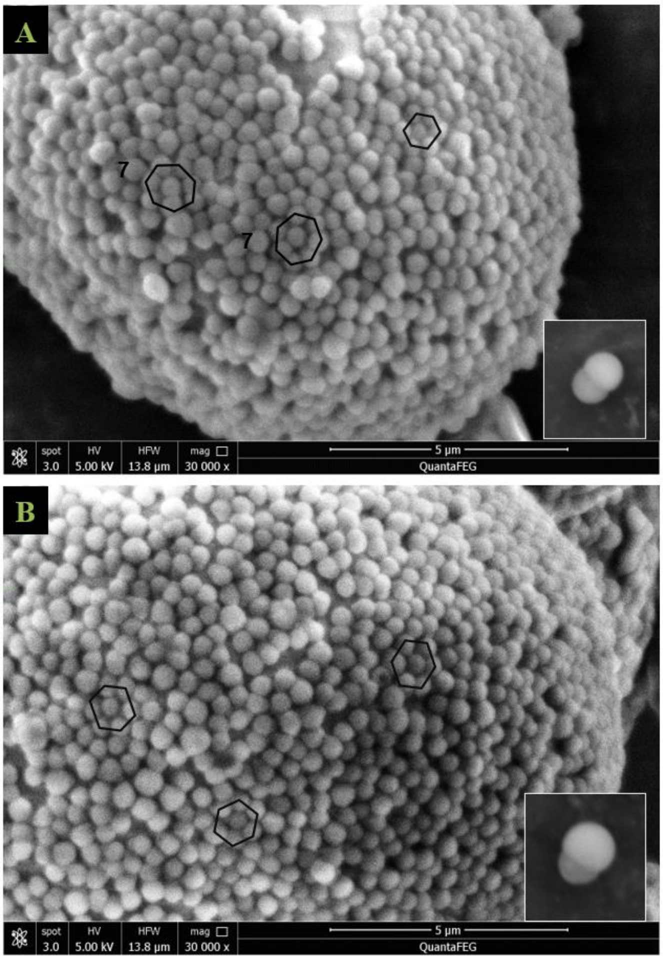

Figure S9. SEM images of Au sputtered colloidosomes with inserts of the JNP-bPEI (not to scale) used for their preparation. Colloidosome decorated with (A) JNP-bPEI 2 mL and (B) JNP-bPEI 4 mL.

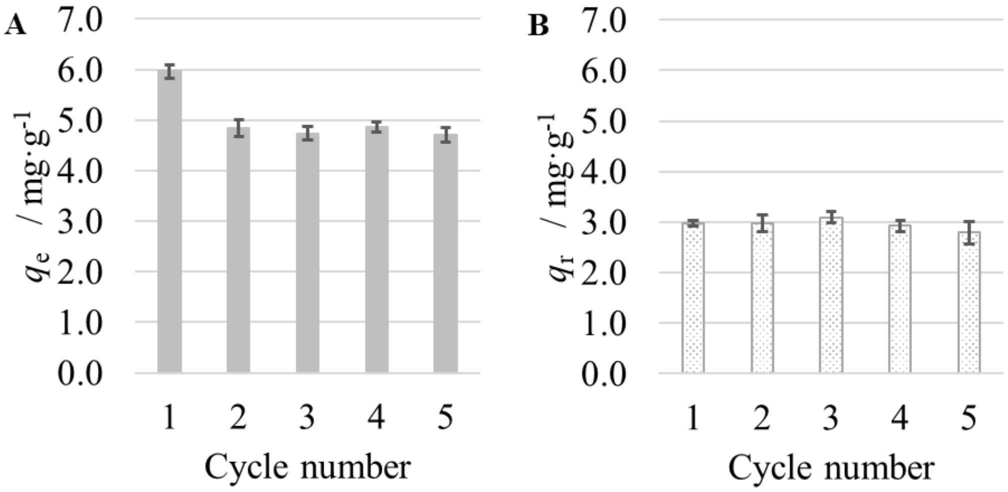

Figure S10. Graphs showing the performance of JNP-bPEI 2 mL after five consecutive (A) extraction and (B) recovery cycles for Cu(II).

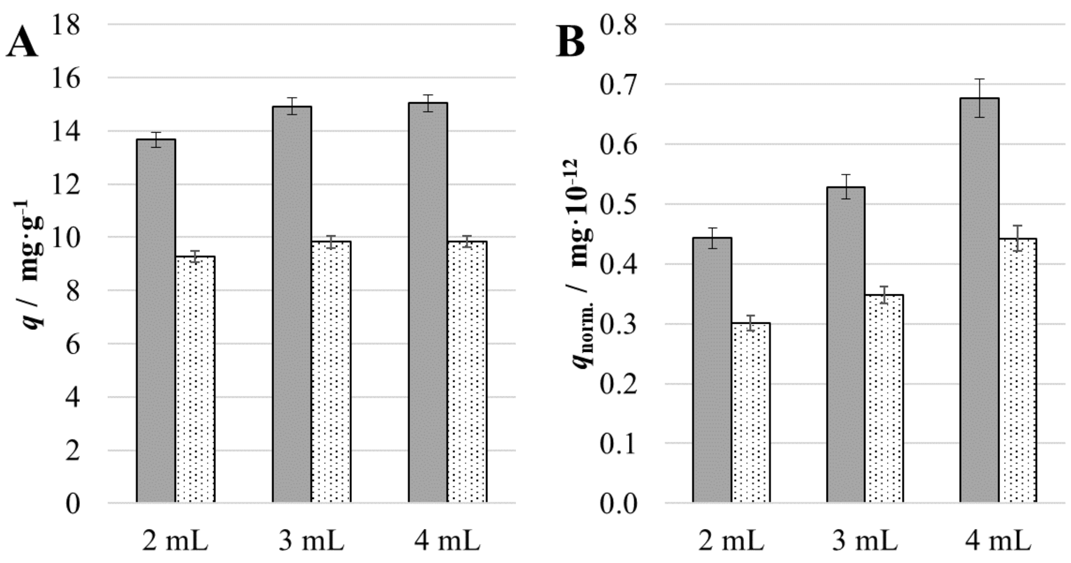

Figure S11. Regular and number-normalized values for Cr(VI) extraction (solid grey) and recovery (dotted) by JNP-bPEI 2 mL, JNP-bPEI 3 mL and JNP-bPEI 4 mL: (A) q_e and q_r and (B) q_{Ne} and q_{Nr}.

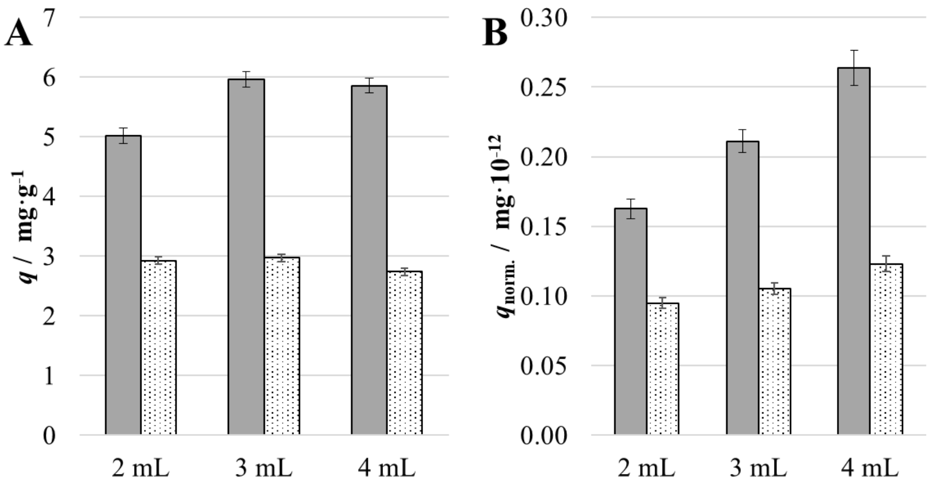

Figure S12. Regular and number-normalized values for Cu(II) extraction (solid grey) and recovery (dotted) by JNP-bPEI 2 mL, JNP-bPEI 3 mL and JNP-bPEI 4 mL: (A) q_e and q_r and (B) q_{Ne} and q_{Nr}.

Table S2. Metal ion extraction and recovery capacities of the homologous series of JNP-bPEI.

JNP	$q_e(Cu^{2+})$ / mg·g^{-1}	$q_r(Cu^{2+})$ / mg·g^{-1}	$q_e(Cr^{6+})$ / mg·g^{-1}	$q_r(Cr^{6+})$ / mg·g^{-1}
JNP-bPEI 2 mL	5.0 ± 0.1	2.9 ± 0.1	13.7 ± 0.3	9.3 ± 0.1
JNP-bPEI 3 mL	6.0 ± 0.1	3.0 ± 0.1	14.9 ± 0.3	9.8 ± 0.1
JNP-bPEI 4 mL	5.9 ± 0.1	2.7 ± 0.1	15.0 ± 0.3	9.8 ± 0.1

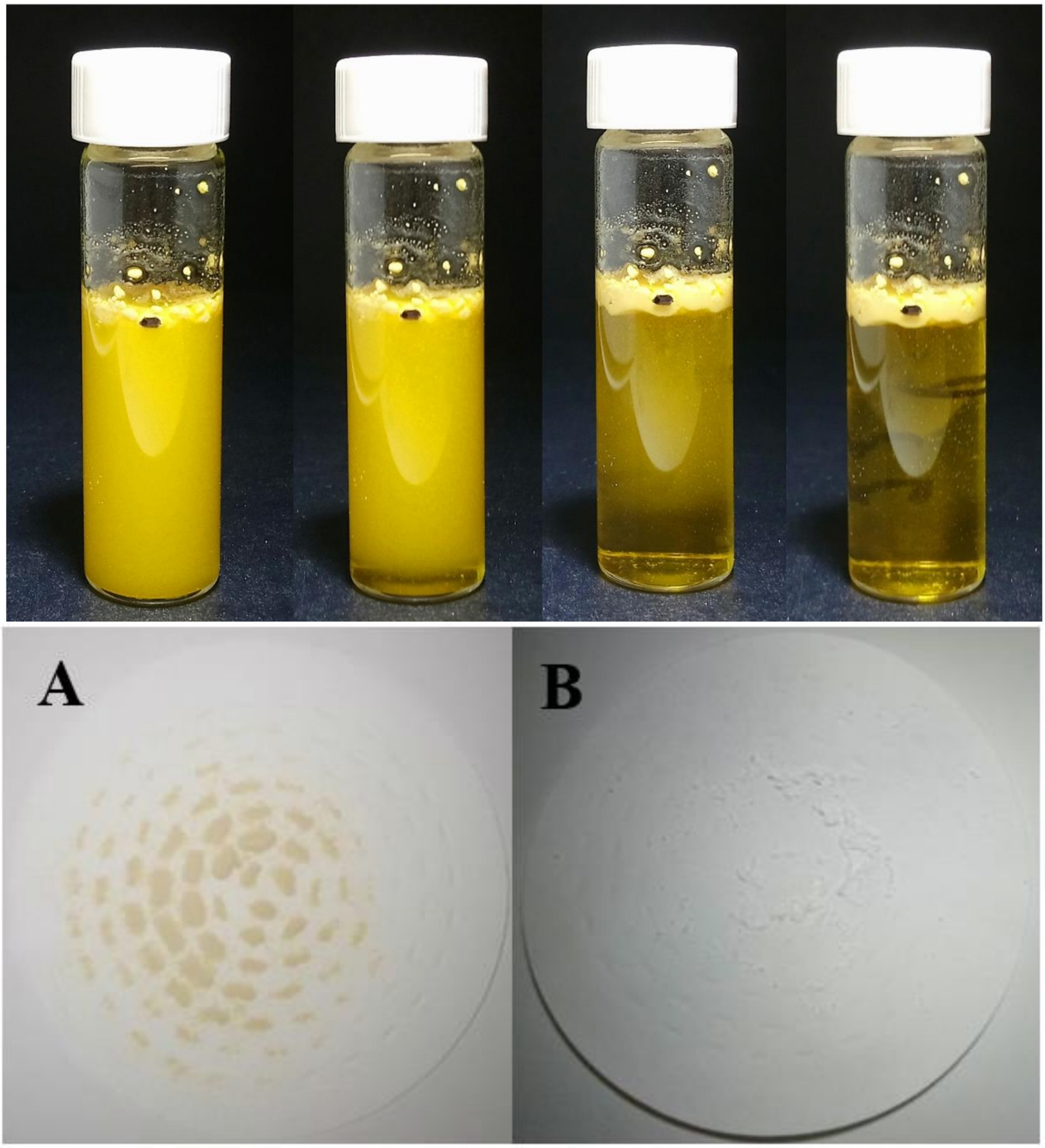

Figure S13. (Top) Sequence of images taken at 10 minute intervals after initial shaking of a 200 mg wax colloidosomes floating above a 5 mL Cr(VI) aqueous solution with a concentration of 10 mmol/L. After 30 minutes the wax colloidosomes can be found floating on the surface, with complete separation from the water phase. (Bottom) (A) Image of filtered and dried colloidosomes after interaction with a Cr(VI) solution overnight and washing with copious amounts of UPW. (B) Image of filtered and dried colloidosomes after interaction with a 0.25% H_2SO_4 solution for metal desorption overnight and washing with UPW.
